# Test–Retest Reliability of the Impact of Vision Impairment–Very Low Vision Questionnaire

**DOI:** 10.1167/tvst.12.6.6

**Published:** 2023-06-12

**Authors:** David J. Fink, Jan H. Terheyden, Susanne G. Pondorfer, Frank G. Holz, Robert P. Finger

**Affiliations:** 1 Department of Ophthalmology, University Hospital Bonn, Bonn, Germany; 2 Department of Ophthalmology, University Medical Center Mannheim, Heidelberg University, Mannheim, Germany

**Keywords:** low vision, patient-reported outcome, questionnaire, IVI–VLV

## Abstract

**Purpose:**

Most patient-reported outcome measures used in ophthalmology show floor effects in a very low vision population, which limits their use in vision restoration trials. The Impact of Vision Impairment–Very Low Vision scale (IVI–VLV) was developed to specifically target a very low vision population, but its test–retest reliability has not been investigated yet.

**Methods:**

The German version of the IVI–VLV was administered twice to patients with stable disease of a low vision clinic. Test and retest person measures of the IVI–VLV subscales were obtained from Rasch analysis. Test–retest reliability was investigated by intraclass correlation coefficients and Bland–Altman plots.

**Results:**

We included 134 patients (72 women, 62 men) at a mean age of 62 ± 15 years. The intraclass correlation coefficients were 0.920 (95% confidence interval, 0.888–0.944) for the activities of daily living and mobility subscale of the IVI–VLV and 0.929 (95% confidence interval, 0.899–0.949) for the emotional well-being subscale. Bland–Altman plots did not indicate any systematic bias. In linear regression analysis, test–retest differences were not significantly associated with visual acuity or administration interval.

**Conclusions:**

Both subscales of the IVI–VLV showed excellent repeatability independent of visual acuity and length of repeat interval. Further validation steps including an assessment of the patient-reported outcome measure's responsiveness are required for use in vision restoration trials.

**Translational Relevance:**

The results support repeated use of the IVI–VLV as a patient-reported end point in future studies in very low and ultralow vision populations.

## Introduction

Restoring vision in people with severe visual impairment or blindness has been a key research goal of generations of ophthalmologists and vision scientists. With the recent technological advances in biomedicine, pharmacology, and electrical engineering, the chances of effective solutions improving sight even in individuals with little or no vision are increasing continuously.^1^ However, regulatory approval of vision restoration technologies requires end points that are capable of reliably capturing even small changes in vision, which is not possible using conventional visual function end points such as visual acuity, visual field parameters, or electroretinography.^2^^,^^3^

Assessments that directly reflect improvements in individuals’ performance of everyday tasks or vision-related quality of life (VRQoL) have thus been suggested as alternative end points in patients with very low and ultralow vision.^4^ Performance-based measures suited for patients with very low or ultralow vision include obstacle courses, which are known to be reproducible between different study sites to only a limited extent,^5^^,^^6^ and specific performance-based tests such as the Instrumental Activities of Daily Living Tool,^7^^,^^8^ Very Low Vision Orientation and Mobility Test Battery,^9^ or the Melbourne Low-Vision ADL-Index.^5^ However, all of the mentioned performance-based measures have the disadvantage of being time and resource intensive. Conversely, instruments assessing VRQoL can be implemented into clinical trials with very little resources particularly if they are self-administered. Most generic VRQoL instruments show noticeable floor effects in a very low or ultralow vision population, which is why specific instruments have been developed.^3^

Of the few instruments specifically targeted at patients with very low or ultralow vision available and thus of interest for future vision restoration trials, the Impact of Vision Impairment-Very Low Vision (IVI–VLV) scale is the only advanced patient-reported outcome measure (PROM) that has been designed to assess VRQoL and functional vision for use in future restoration trials in a very low vision population.^3^ It may further be useful as a patient-relevant end point in future trials on low vision aids, assistive devices, and in the context of eHealth.

Other similarly advanced PROMs were either only designed to assess functional vision but not VRQoL, such as the VALVVFQ or the ABS/AI, or target different patient groups as the ULV-VFQ ultra low vision patients or the PedEyeQ children with low vision.^10^^–^^14^ The IVI–VLV was developed initially based on qualitative work involving 603 legally blind people and quantitative assessments, including internal consistency (person reliability 0.85 and 0.83 for the ADLMS and EWB subscales), and construct validity (association between ADMLS subscale person measures and level of visual impairment [*P* = 0.003]; between EWB subscale person measures and anxiety/depression [*P* = 0.008]). Moreover, the IVI–VLV has been shown to be content valid and well-interpretable and has only limited floor and ceiling effects in the target group.^10^

Nonetheless, the repeatability of the IVI–VLV has not been assessed previously, which will be an important prerequisite for repeated administrations of the PROM during clinical trials. To fill this gap, we have assessed the test–retest reliability of the IVI–VLV.

## Methods

### Study Design

Participants 18 years and older were recruited from the outpatient low vision clinic at the Department of Ophthalmology, University Hospital Bonn, Germany. The Institutional Review Board approved the study (approval ID: 130/16 and 296/18). We adhered to the principles of the Declaration of Helsinki and written informed consent was obtained from all participants before study inclusion. Inclusion criteria were stable chronic eye conditions and the ability to understand, answer, and read in German. Exclusion criteria were acute deterioration or amelioration of visual function within the timeframe of the study or within 6 weeks before study start, illiteracy, cognitive impairment, insufficient knowledge of German language, and intraocular surgery 3 months before or in between the different PROM administrations. Inclusion and exclusion criteria were applied based on patient files or self-reports in cases where no recent examinations in the recruiting institution took place.

### IVI–VLV Questionnaire

The IVI–VLV was developed as a measure of VRQoL with patients with very low vision, experts, and input from the literature and is derived from the original IVI questionnaire, focusing on aspects more relevant in the context of very low and ultralow vision.^10^ It contains 28 items that are grouped into two subscales—16 items measuring activity and mobility of daily living (ADLM) and 12 items evaluating emotional well-being (EWB). All items employ a common rating scale with four response options each (ADLM from not at all to a lot; EWB from not at all to a lot of the time), as well as an additional option phrased “Do not do this for other reasons.” The full version with exact phrasing has been previously published.^10^ Its English language version was developed in 603 individuals with a better eye visual acuity of less than <20/200 or a binocular visual field of 10° or less that, in the major proportion of participants, was due to AMD, retinal dystrophies, or glaucoma.^10^

For this study, we translated and culturally adapted the IVI–VLV into German, following a methodology recommended by the International Society for Pharmacoeconomics and Outcomes Research.^15^ In brief, two forward translations of the English IVI–VLV were developed by professional medical translators. Both were condensed into a single German version that was then translated back into English by two independent translators blinded to the original versions. The developer (R.P.F.) reviewed and provided input into the final translation. Finally, the German version was proofread by a medical translator and underwent cognitive debriefing in five patients. This version was then administered to the participants of our study. In our study, the IVI–VLV was administered in a calm environment by one interviewer specifically trained for the study in person or if necessary via phone. The retest administration took place within on average 19 ± 16 days (range, 1–103 days) after the initial questionnaire administration. Visual acuity and other clinical data were available from recent visits at the outpatient department. Additionally, we conducted Goldman perimetry with patients where we suspected visual impairment owing to visual field restriction. The evaluation was performed according to the recommendations of the International Council of Ophthalmology using the Functional Field Score.^16^ All acquired data were stored in pseudonymized form following the institution's data security guidelines.

### Scoring

We used Rasch analysis to calculate person measures (expressed in logits) from the original responses, using a partial credit model. Rasch analysis assumes that all items of the same subscale form an underlying latent trait, which makes it more robust to outliers than classical test theory.^17^ We identified misfitting items (outfit outside 0.6–1.4) based on mean squared fit statistics and removed misfitting item responses as well as misfitting items as appropriate.^18^ We then investigated threshold ordering of the rating scale and collapsed response categories if indicated. We interpreted differences of more than 1.0 logits between mean person and mean item measures as indicators of mistargeting.^19^ Last, we assessed differential item functioning for the type of interview (face-to-face or via phone) for each item, with contrasts of greater than 1.0 indicating differential item functioning.^20^ Rasch analysis was conducted using WINSTEPS software (Chicago, IL, version 3.92.1).^21^

### Statistical Analyses

Data were analyzed descriptively followed by the assessment of test-retest reliability, using intraclass correlation coefficients (ICCs) and Bland–Altman plots. We conducted linear regression analysis with mean differences between test and retest administrations as the dependent variable and visual acuity, as well as administration interval as independent variables, to adjust for variance of these factors in our dataset. We assessed the standard error of measurement (SEm) by calculating the standard deviation out of the difference between test and retest and dividing it by √2.^22^^,^^23^ All statistical analyses were performed using SPSS (version 25, IBM, Armonk, NY) and RStudio (version 1.4.1717, RStudioTeam, Boston, MA).

## Results

The IVI–VLV was administered to 134 patients (72 women, 62 men) with a mean age of 62 ± 15 years. The mean best-corrected visual acuity in the better eye was 0.8 ± 0.7 logarithm of the minimum angle of resolution (LogMAR) (Table 1). Interviews were conducted in person (32%) or via phone (68%). Goldman perimetry was performed in 47 participants. Nineteen participants had a functional field score of 50 or less, which corresponds with a binocular visual field of 10° or less.^16^

**Table 1.** Participant Characteristics (*n* = 134)

Characteristic	Mean ± SD or *n* (%)
Age (in years)	62.1 ± 14.9
Sex	
Male	62 (46.3%)
Female	72 (53.7%)
VA (logMAR)	2.3 ± 1.0
VA better eye	0.8 ± 0.7
VA worse eye	1.2 ± 0.8
Distribution VA (logMAR) of the better eye	
≥1.0	29 (21.6%)
≥0.5, <1.0	35 (26.1%)
<0.5	70 (52.2%)
Distribution of the FFS (*n* = 47)	
>50	28 (20.9%)
≤50	19 (14.2%)
Perimetry not performed	87 (64.9%)
Time between interviews (in days)	19.3 ± 15.9
≤10	32 (23.9%)
11–20	59 (44.0%)
21–30	28 (20.9%)
>30	15 (11.2%)
Eye condition	
Age-related macular degeneration	31^*^
Glaucoma	16^*^
Rod–cone dystrophies	13
Cone/cone–rod dystrophies	7
Stargardt disease	8
Other retinal/chorioretinal dystrophies	10^*^
Degenerative myopia	12^*^
Pseudoxanthoma elasticum	6^*^
Mac Tel type 2	4
Other retinal pathologies	26^*^
Other optic nerve pathologies	7^*^
Other	12

FFS = Functional Field Score; SD = standard deviation; VA = visual acuity.

* Does not add up to 134 because 18 participants had >1 diagnosis contributing to vision.

In the Rasch analysis of the complete IVI–VLV containing both subscales, 25 individual responses from 4 items showed misfit to the model and were dropped to improve measurement precision of the latent trait model. After this step, no further item showed misfit. Person reliability and person separation were within acceptable limits (Table 2). Three items (no. 6, 12, 22) had disordered thresholds. Therefore, we collapsed two categories of items 12 and 22 (some of the time and a little of the time) and dropped one response to item 6, resulting in the thresholds of all items being ordered. No item had contrasts indicative of differential item functioning for the type of interview.

**Table 2.** The Fit Parameters of the ADLMS and EWB Compared With the Rasch Model

Parameters	Rasch Model Requirements	IVI–VLV Complete	IVI–VLV ADLM	IVI–VLV EWB
Misfitting items, n		2 (No. 16–12, No. 17–9; No. 22–3)	0	1 (No. 20)
PSI	>2.0	3.31	2.53	2.12
PR	>0.8	0.92	0.86	0.82
Difference in person and item mean	<1.0	0.84	0.87	0.9
First contrast in principal component analysis of residuals	<2.5	3.0	2.4	1.7

PR = Person reliability; PSI = Person separation index.

ICCs were 0.920 (95% confidence interval [CI], 0.888–0.944) for the ADLM subscale and 0.929 (95% CI, 0.899–0.949) for the EWB subscale. Separate ICCs for subgroups of individuals with a visual acuity of 1.0 logMAR or greater and a Functional Field Score of 50 or less showed comparable results (Table 3). The mean differences between test and retest assessments were −0.11 ± 0.73 for the ADLM subscale and 0.11 ± 0.67 for the EWB subscale. In linear regression analysis, test–retest differences were not significantly associated with visual acuity or the administration interval in either subscale (*P* ≥ 0.563 and 0.156, respectively). Last, we did not notice any systematic patterns in Bland–Altman analysis (Fig.).

**Table 3.** ICC: Single-Rating, Absolute Agreement Two-Way Random Effects Model

		95% Confidence Interval	
Single Measures	Intraclass Correlation	Lower Bound	Upper Bound
ADLM			
Full cohort	0.920	0.888	0.944
VA logMAR ≥ 1.0 or FFS ≤ 50	0.932	0.870	0.965
VA logMAR <1.0 and FFS > 50	0.914	0.871	0.943
EWB			
Full cohort	0.929	0.899	0.949
VA logMAR ≥ 1.0 or FFS ≤ 50	0.931	0.869	0.964
VA logMAR <1.0 and FFS > 50	0.926	0.889	0.951

FFS = Functional Field Score; VA = visual acuity.

**Figure.**
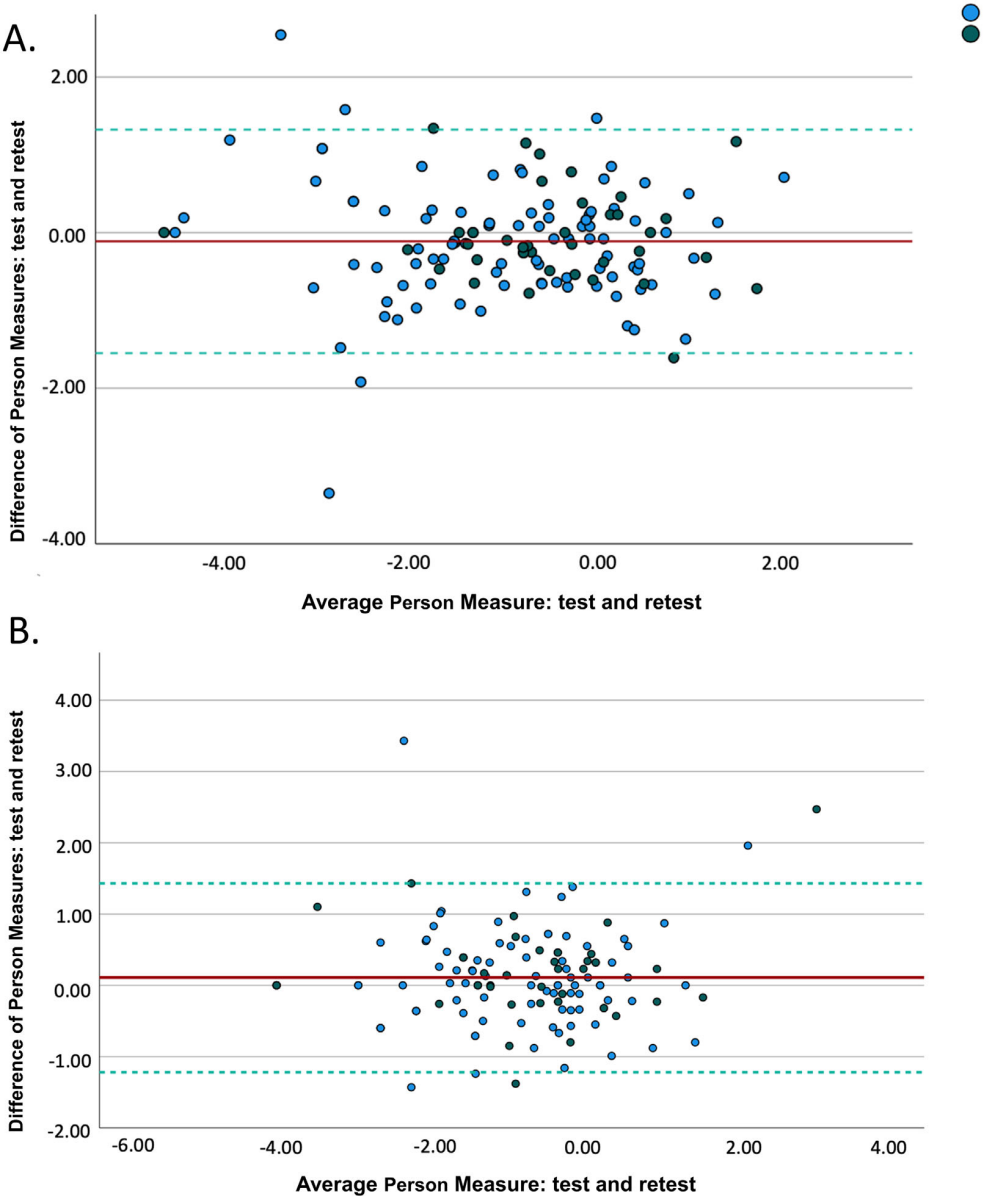
Bland–Altman plots of the activity of daily living and mobility (ADLM) subscale **(A)** and the emotional well-being (EWB) subscale **(B)**. *Green dots*, visual acuitiy of ≥1.0 logMAR or functional field score of ≤50; *blue dots*, visual acuity of <1.0 logMAR and functional field score of >50.

The described results were also supported by an analysis of the unprocessed dataset (ICC, 0.919 [95% CI, 0.885–0.942] for the ADLM subscale; ICC, 0.935 [95% CI, 0.909–0.954] for the EWB subscale).

The SEm of the Person measures was 0.48 for the EWB and 0.52 for the ADLM subscale. For the EWB subscale, the SEm accounts for 5.6% of the entire scale (−4.17 to 4.37), for the ADLM subscale the SEm represents 5.2% of the entire scale (−4.71 to 4.70).

## Discussion

Regulators suggest the ICC as a key readout in test–retest reliability assessments of PROMs.^4^ Our study indicates excellent test–retest agreement of the IVI–VLV with ICCs of greater than 0.9 and no proportional differences. The lower limits of the confidence interval of the ICCs and additional read-outs of our study also support the IVI–VLV being highly test–retest reliable. Additional analyses suggested that the test–retest reliability of the IVI–VLV was independent of visual acuity and the retest administration interval. Thus, the IVI–VLV can be reliably used in settings where multiple administration over time are conducted.

The IVI–VLV was developed based on the same item pool from which the IVI scale, a PROM targeting individuals with less severe visual impairment than the IVI–VLV, was constructed. Similar to the IVI–VLV, the IVI is a validated PRO outcome instrument that captures functional vision and VRQoL. In our study, the IVI–VLV had a test-retest reliability comparable to the IVI (IVI: ICC, 0.912–0.938 [Terheyden J. H. & Ost R. et al. unpublished data, March 2023]; IVI–VLV: ICC, 0.920–0.929). The results of our study are also in line with repeatability data of the veterans administration Low-Vision Visual functioning Questionnaire (LV-VFQ), another PRO measure targeted at a low vision population (VA-VFQ: ICC, 0.84).^12^ Our retest sample was larger than the sample reported by Stelmack et al. for the LV-VFQ, which consisted of only 30 participants. The LV-VFQ was developed to evaluate low vision outcomes in rehabilitation services, whereas the development of the IVI–VLV was with the aim to be used in clinical trials restoring vision. Both questionnaires are targeting a low to very low vision population. The LV-VFQ targets especially the assessment of functional vision in rehabilitation services, whereas the IVI–VLV aims to evaluate visual impairment by a broader scope by assessing functional VRQoL and health-related quality of life in no fixed context.

The Rasch model that we have applied in our studies has the advantages of being comparable with the initial validation study of the IVI–VLV and being widely accepted in the analysis of PROMs, but also has disadvantages, which includes the requirement of ordered thresholds.

The main strengths of our study include that specifically trained interviewers recorded the data following a standardized interview guideline as well as our large sample size compared to other studies assessing test–retest reliability in low vision PROMs.^23^^,^^12^^,^^24^ We used modern statistical techniques which supported our interpretation of the main findings. Use of latent trait models enabled us to evaluate test–retest reliability on a more comprehensive and advanced basis than with classical test theory.^3^

The main limitation of our study is its relatively heterogeneous sample. The target group of the IVI–VLV was initially defined by a visual acuity of 1.0 logMAR or greater or visual field deficit; we enriched our sample with better seeing individuals.^10^ However, visual acuity was not significantly associated with test–retest differences so that the effect appears to be negligible and in a subgroup analysis in 39 study participants with a best-corrected visual acuity of less than 20/200 in the better eye or a Functional Field Score of 50 or less repeatability was comparable with the overall cohort (Table 3).

It is commonly recommended to administer test and retest assessments of PROMs within 7 to 14 days.^25^ The average readministration interval was 19 ± 16 days, with 89% of the retest assessments performed within the first 30 days after the initial test. Because the vision-relevant eye conditions in our cohort worsen very slowly over time, we do not consider shorter readministration intervals crucial for the target population of the IVI–VLV. Regression analyses supported this and retest intervals were not significantly associated with differences between test and retest administrations of the IVI–VLV. All administrations were conducted by the same specifically trained interviewer but both face-to-face administration and administration via phone were allowed because some participants could not attend face-to-face interviews owing to long travel distances. However, we did not find repeatability of the IVI–VLV to be noticeably different between those interviewed face-to-face versus phone interviews. When used in a multi-language setting, different language version of the IVI–VLV may require further assessment of cultural differences between language versions, which was not covered by our study.

Overall, the IVI–VLV proved excellent repeatability, which was independent of the level of visual impairment and repeat interval. The results of our study support further use of the IVI–VLV in a very low and ultralow vision population, such as in the context of vision restoration trials.

## Supplementary Material

Supplement 1
