# Supplementary material for: Test–Retest Reliability of the Impact of Vision Impairment–Very Low Vision Questionnaire
Source: Transl Vis Sci Technol. 2023 Jun 12;12(6):6. doi: 10.1167/tvst.12.6.6 (PMC10266551; doi:10.1167/tvst.12.6.6)
Supplement: Supplement 1 [file tvst-12-6-6_s001.pdf]

Supplement

Supplementary Table 1. Overview of Impact of Vision Impairment-Very Low Vision item measures and standard errors*

IVI-VLV Item Number	Item Measures	Standard Error
Activities of Daily Living and Mobility		
1	0.56	0.12
2	0.46	0.12
3	0.09	0.11
4	-0.035	0.11
5	-0.53	0.11
6	1.38	0.16
7	-0.89	0.11
8	-0.71	0.12
9	-0.60	0.11
10	-0.30	0.11
11	0.75	0.13
12	0.34	0.12
13	0.65	0.12
14	-0.60	0.1
15	-0.52	0.1
16	-0.86	0.1
Emotional Well Being		
17	0.79	0.13
18	-0.71	0.10
19	0.63	0.12
20	-0.19	0.10
21	-0.10	0.10
22	0.76	0.14
23	0.46	0.12
24	1.30	0.16
25	-0.78	0.1
26	-1.13	0.10
27	1.00	0.14
28	-1.49	0.11

*For the full instrument, please see Finger et al., Invest Ophthalmol Vis Sci 2014

```
MEASURE PERSON - MAP - ITEM
            <more>|<rare>
    3             +
                  |
                  |
               X  |
                  |
                  |
                  |
                  |
    2             +
                  |
                  |
                  |
                  |T
             XXX T|  6
               X  |  24
              XX  |
    1             +  27
                  |
               X  |S 11 22 17
               X  |  13 19
              XX  |  1 2 23
          XXXXXX S|  12
         XXXXXXX  |
             XXX  |  3
    0    XXXXXXX  +M 14
         XXXXXXX  |  21
        XXXXXXXX  |  10 20
             XXX  |  4
       XXXXXXXXX  |  5 15
           XXXXX  |  9
        XXXXXXXX M|S 8 25 18
             XXX  |  16 7
   -1       XXXX  +
           XXXXX  |  26
             XXX  |
          XXXXXX  |
        XXXXXXXX  |T 28
              XX  |
            XXXX  |
               X S|
   -2     XXXXXX  +
             XXX  |
              XX  |
               X  |
              XX  |
              XX  |
                  |
             XXX  |
   -3            T+
               X  |
               X  |
                  |
                  |
                  |
                  |
               X  |
   -4         XX  +
            <less>|<freq>
```

Supplementary Figure 1. Wright Map of the IVI-VLV: Persons n=134, items n=28 performed in WINSTEPS 3.92.1

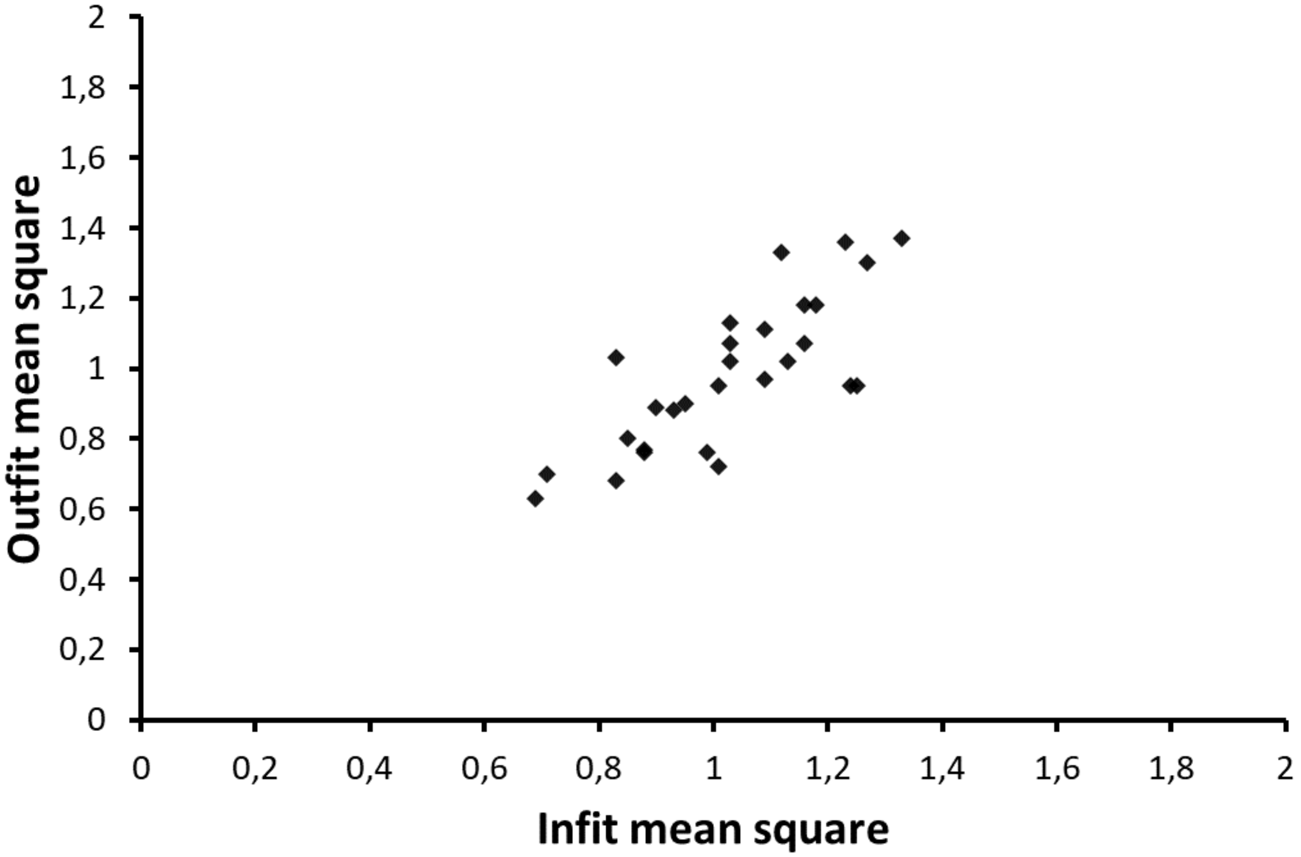

Supplementary Figure 2. Infit and Outfit mean square values of the 28 items of the IVI-VLV
